# Draft Whole-Genome Sequence of *Lactobacillus fermentum* LfQi6, Derived from the Human Microbiome

**DOI:** 10.1128/genomeA.00423-15

**Published:** 2015-05-14

**Authors:** Bobban Subhadra, Jill Krier, Karina Hofstee, Nicholas Monsul, Eva Berkes

**Affiliations:** Quorum Innovations LLC, Sarasota, Florida

## Abstract

We report a 2.21-Mbp draft whole-genome sequence of *Lactobacillus fermentum* Qi6 (LfQi6). This strain demonstrates activity against pathogenic biofilms, enhances the skin barrier, and upregulates innate immune defenses. The genome sequence information of this strain will help to identify molecules that hold promise for the discovery of novel therapeutics for dermatological disorders.

## GENOME ANNOUNCEMENT

The human microbiome’s role in modulating health and disease is a growing theme in medical research (1–3). We isolated LfQi6 from human microbiota that showed strong biofilm formation. Bioactives from LfQi6 have novel bimodal biofilm modulation; that is, they inhibit and detach pathogenic biofilm while enhancing commensal resident biofilm on skin. LfQi6 promoted skin barrier and immune functions by upregulating the expression of the essential skin barrier homeostatic protein, filaggrin, in human skin. These studies suggest that this strain holds the potential for discovering novel molecules for dermatological disorders involving skin-barrier deficiencies and dysbiosis, such as atopic dermatitis, rosacea, ichthyosis, and acne vulgaris. Therefore, complete genome sequencing of this strain was undertaken.

LfQi6 genomic DNA (4 ng/µl) was used for the indexed Nextera XT sequencing library with 300- to 600-bp insert size and to prepare a Nextera mate-pair (4 to 10 kb) sequencing library. Pooled paired-end libraries were loaded onto MiSeq Flow Cell to generate 2 × 150-bp paired-end reads with average coverage of 250× per genome. Pooled libraries were loaded on the MiSeq platform and sequenced with 2 × 150-bp paired-end reads to obtain at least 50× coverage of the genome. Illumina sequence reads were demultiplexed, raw reads were converted into .fastq format, and low-quality and short reads were filtered out. MiSeq Reporter software filtered and demultiplexed the sequencing reads, giving 2,344,249 paired-end (2 × 150-bp) and 940,098 mate-pair reads (2 × 150-bp). The filtered reads were *de novo* assembled to generate contigs, combining paired-end and mate-pair read data. Sequences were trimmed using trim galore software to give 2,309,153 paired-end and 562,149 mate-pair reads and *de novo* assembled using Velvet and SPAdes. Assemblies with the largest *N*_50_ and/or contig size were BLASTed against GenBank. BLAST results show that the sequenced sample has 99% identity with the three published *L. fermentum* genomes at the nucleotide level (4–6). Further BLAST searches suggest the possible presence of repeats and genomic rearrangements. The best contig assembly was scaffolded based on the *L. fermentum* IFO 3956 genome (AP008937) (4), using Scaffold_builder and a *de novo* approach using SSPACE. Since the *de novo* approach yielded better results, all downstream bioinformatics analyses were based on the *de novo* scaffold sequences.

The final assembly is 2.21 Mbp with 37 scaffolds; the genome *N*_50_ is 252,403; the largest scaffold is 484,720 bp with a mean scaffold of 59,600 bp. The assembled genome was annotated with 2,012 coding sequences and 64 RNA genes. For the function-based comparison, 1,070 coding sequences were present in both *L. fermentum* IFO 3956 and the LfQi6 genome, with 64 coding sequences present only in *L. fermentum* IFO 3956 and 101 coding sequences present only in the LfQi6 genome. We identified unique protein-coding sequences in the LfQi6 genome, including fibronectin-binding proteins, the cold shock protein CspB, the GTP-binding protein HflX, the Clp protease-like protein, murein hydrolases, and several paralogs of DNA-repair proteins. Detailed characterization of these proteins in biofilm modulation, skin barrier, and immune functions are under way.

### Nucleotide sequence accession number. 

This whole-genome shotgun project has been deposited in GenBank under the accession number LAIK00000000.
